# Development of ytterbium-doped oxyfluoride glasses for laser cooling applications

**DOI:** 10.1038/srep21905

**Published:** 2016-02-26

**Authors:** Kummara Venkata Krishnaiah, Elton Soares de Lima Filho, Yannick Ledemi, Galina Nemova, Younes Messaddeq, Raman Kashyap

**Affiliations:** 1 Department of Engineering Physics, Polytechnique Montréal, 6079, Station Centre-ville, Montréal H3C 3A7, Canada; 2 Centre d’Optique, Photonique et Laser, 2375 Rue de la Terrasse, Université Laval, Québec G1V 0A6, Canada; 3 Department of Electrical Engineering, Polytechnique Montréal, 6079, Station Centre-ville, Montréal H3C 3A7, Canada

## Abstract

Oxyfluoride glasses doped with 2, 5, 8, 12, 16 and 20 mol% of ytterbium (Yb^3+^) ions have been prepared by the conventional melt-quenching technique. Their optical, thermal and thermo-mechanical properties were characterized. Luminescence intensity at 1020 nm under laser excitation at 920 nm decreases with increasing Yb^3+^ concentration, suggesting a decrease in the photoluminescence quantum yield (PLQY). The PLQY of the samples was measured with an integrating sphere using an absolute method. The highest PLQY was found to be 0.99(11) for the 2 mol% Yb^3+^: glass and decreases with increasing Yb^3+^ concentration. The mean fluorescence wavelength and background absorption of the samples were also evaluated. Upconversion luminescence under 975 nm laser excitation was observed and attributed to the presence of Tm^3+^ and Er^3+^ ions which exist as impurity traces with YbF_3_ starting powder. Decay curves for the Yb^3+^: ^2^F_5/2_ → ^2^F_7/2_ transition exhibit single exponential behavior for all the samples, although lifetime decrease was observed for the excited level of Yb^3+^ with increasing Yb^3+^ concentration. Also observed are an increase in the PLQY and a slight decrease in lifetime with increasing the pump power. Finally, the potential of these oxyfluoride glasses with high PLQY and low background absorption for laser cooling applications is discussed.

Ytterbium (Yb^3+^)-doped glasses have been widely investigated for their potential in solid-state lasers^1^,^2^, downconversion^3^, upconversion^4^, light emitting diodes^5^, athermal lasers and more recently in solid-state laser induced cooling^6^. Basic requirements for laser cooling applications include materials of low phonon energy, low background absorption, high purity and photoluminescence quantum yield (PLQY)^7^.

Laser induced cooling based on anti-Stokes fluorescence was first proposed by Pringsheim^8^ and experimentally demonstrated for solids by Epstein *et al.* in Yb^3+^-doped ZBLANP glass^6^. Since 1995 laser cooling based on anti-Stokes fluorescence have been reported in a wide variety of low phonon energy host materials^9^,^10^,^11^,^12^ doped with Yb^3+^, Er^3+^ and Tm^3+^ ions^12^,^13^,^14^,^15^. Studies have been focused on the Yb^3+^ (4f^13^) ion as it has a very simple energy level structure consisting of only two manifolds, the ground (^2^F_7/2_) and excited (^2^F_5/2_) states which are well separated by about 10,000 cm^−1^. Yb^3+^-doped laser materials can be efficiently pumped by high-power commercially available diode lasers with wavelength in the range of 0.9–1.1 μm. Laser operation takes thus place in the 1.0 μm wavelength region close to the 1.06 μm wavelength laser line of Nd^3+^ ion. Efficient lasing is possible in Yb^3+^-doped materials because of small quantum defect (the energy difference between pump and lasing photons) which is not only the primary source of heating but also the source of anti-Stokes fluorescence for cooling^1^,^9^.

The PLQY of rare earth (RE)-doped solids is strongly influenced by the maximum phonon energy of the host, which determines the non-radiative relaxation rate. Fluoride glasses are thus favorable hosts for achieving higher PLQY owing to their low phonon energy (~580 cm^−1^, for the ZBLAN fluorozirconate glass) when compared with traditional oxide glasses (~1100 cm^−1^, for silicate glass). However, it is difficult to use them for practical applications due to their limited mechanical and chemical resistance. On the other hand, oxide glasses are usually preferred despite their higher phonon energy as they possess excellent chemical and mechanical properties. Oxyfluoride glasses based on heavy metal fluorides and silicates may surpass oxide and fluoride glasses by combining their advantageous properties such as low phonon energy, low melting point, high chemical durability and mechanical resistance, giving rise to unique materials with superior optical properties for a wide range of applications in photonics^9^,^14^,^15^,^16^,^17^,^18^. In addition, ultra-transparent glass-ceramics containing low phonon energy fluorite nanocrystals could also be produced under appropriate heat-treatment applied to as-made oxyfluoride glasses^19^. Oxyfluoride glasses and glass-ceramics may also be suitable for non-linear optical applications. As reported in the literature^20^, nano-glass-ceramics exhibiting a non-linear refractive index (*n*_2_ = 6.69 × 10^14^ cm^2^/W) about two times larger than that of their parent glasses (*n*_2_ = 3.23 × 10^14^ cm^2^/W) were obtained.

The motivation of our work is to develop low phonon energy oxyfluoride glasses for laser cooling applications. Glassy materials indeed exhibit various advantages over crystals such as ease of fabrication, capability of scaling-up and thus cost-effective production. Here, heavily Yb^3+^-doped oxyfluoride glasses belonging to the SiO_2_-Al_2_O_3_-PbF_2_-CdF_2_-YF_3_ vitreous system were prepared and characterized. Glass transition and crystallization temperatures, thermal stability against crystallization and thermal expansion coefficient were determined by thermal analysis. The PLQY was then evaluated for all the samples by using a pump wavelength at 920 nm from a Ti: sapphire laser and measuring the emission spectra with an integrating sphere coupled to an optical spectrum analyzer. The present study aimed at optimizing the Yb^3+^ ion concentration in order to obtain high PLQY and low background absorption. To the best of our knowledge, this is the first spectroscopic investigation report on these oxyfluoride glasses for laser cooling applications. The obtained results were compared with those reported on Yb^3+^: ZBLANP glass^6^ and Yb^3+^: YAG crystal^12^.

## Theory

**Figure 1:**
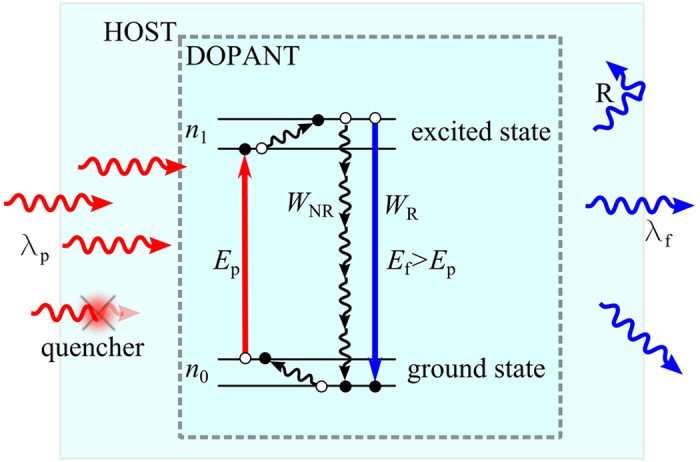
Scheme of laser cooling cycle in Yb^3+^-doped systems. Here *λ*_p_ is the pump wavelength, *λ*_f_ is the fluorescence wavelength, *E*_p_ is the pump energy, *E*_f_ is the fluorescence energy, *R* is the reabsorption, and *W*_r_ is the radiative and *W*_nr_ is the non-radiative decay rates. If impurities (*quencher*: transition metal ions and other impurities) present in the glass matrix (*host*) absorb the pump laser, then luminescence quenching occurs, leading to heating of the sample.

Laser cooling process in RE-doped host, which is based on anti-Stokes fluorescence, is illustrated in Fig. 1. The cooling efficiency of the sample can be described as:


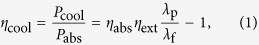



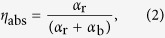


where *η*_abs_ is the absorption efficiency which includes the resonant, *α*_r_ and background absorption, *α*_b_.


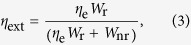


is the external PLQY, where *η*_e_ is the fluorescence escape efficiency, which has been investigated in ref. 21. It also depends on the refractive index and shape of the sample. *W*_r_ and *W*_nr_ are the radiative and non-radiative decay rates, respectively. The fluorescence escape efficiency depends not only on the refractive index but also on the shape of the sample. As can be seen in Eq. (1), only RE-host combinations satisfying the inequality, *W*_nr_ ≪ *W*_r_ are suitable for laser cooling by anti-Stokes fluorescence. The mean fluorescence wavelength, *λ*_f_ can be calculated as:


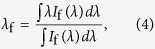


where *I*_f_(*λ*) is the measured emission spectrum without using an integrating sphere.

The external PLQY (*η*_ext_) can be expressed as the ratio of the number of emitted photons and the number of absorbed photons^22^:


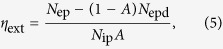


where *N*_ep_ is the number of emitted photons from the sample when the excitation beam is directed onto the sample, *A* is the absorption coefficient, *N*_ip_ is the number of incident photons detected without the sample and *N*_epd_ is the number of emitted photons by the sample with the interaction of diffused light. In addition to Eq. (5) for evaluating the PLQY, we propose another relation to assess the PLQY.


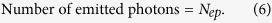



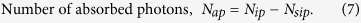


By combining the above two relationships (6) and (7), we can get:


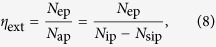


where *N*_ap_ is the number of absorbed photons when the sample is excited directly with the laser in the integrating sphere, *N*_ip_ is the number of incident photons collected without the sample in the integrating sphere and, *N*_sip_ is the number of scattered incident photons detected when the sample is inside the integrating sphere and the beam is directed on it.

## Results and Discussion

### Thermal and thermo-mechanical properties

**Figure 2:**
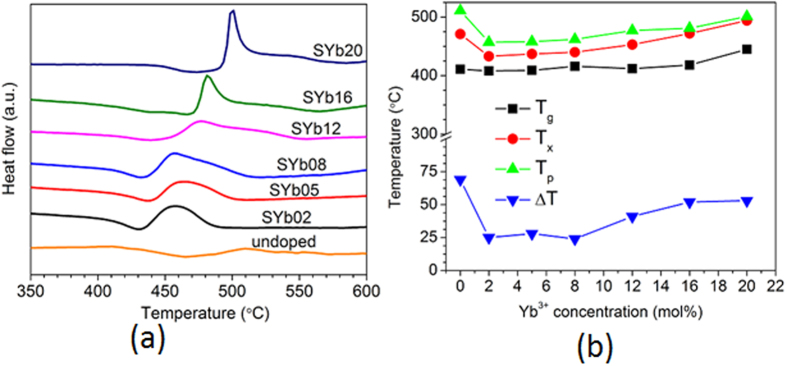
(**a**) DSC traces of the undoped and Yb^3+^-doped 30SiO_2_-15Al_2_O_3_-(29-*x*)CdF_2_-22PbF_2_-4YF_3_-*x*YbF_3_ oxyfluoride glasses as a function of Yb^3+^ concentration (*x*). The thermograms were vertically shifted for better comparison. (**b**) Variation of *T*_g_, *T*_x_, *T*_p_ and Δ*T* of the samples under study as a function of Yb^3+^ concentration.

The thermal and thermo-mechanical properties of the proposed cooling material are essential for investigating the integration of numerous optical parameters of an optical cooler. The DSC traces recorded on the 30SiO_2_-15Al_2_O_3_-(29-*x*)CdF_2_-22PbF_2_-4YF_3_-*x*YbF_3_ (mol%) glasses for various Yb^3+^ ion concentrations are shown in Fig. 2(a). The glass transition temperature (*T*_g_, ±2 °C), the onset temperature of crystallization (*T*_x_, ±2 °C) and peak crystallization temperature (*T*_p_, ±1 °C) were determined from the thermograms as well as the corresponding glass thermal stability against crystallization criterion (Δ*T* = *T*_x_ − *T*_g_, ±4 °C). Among the Yb^3+^-doped samples, a slight increase of their glass characteristic temperatures *T*_g_, *T*_x_ and *T*_p_ is observed in Fig. 2(a,b) with increasing Yb^3+^ concentration. First, the increase of glass transition temperature (from 412 to 445 °C) with increasing Yb^3+^ content (from 0 to 20 mol%) shows here that the Yb^3+^ ions are well incorporated into the glass network. Whereas addition of RE ions like Yb^3+^ into a glassy material usually tends to decrease its stability by altering its network reticulation (and thus decreasing its *T*_g_), it seems here that the addition of Yb^3+^ reinforces the glass network. The heavy level of doping attained (up to 20 mol%) supports this assumption. Further investigation is required to understand the structural role played by Yb^3+^ in such heavily doped glasses.

A progressive shifting towards higher temperature of the crystallization peak can be observed on the DSC traces (Fig. 2(a)) with increasing Yb^3+^ concentration. This slight increase of *T*_x_ and *T*_p_ can be directly correlated with the strengthening of the glass network after the replacement of Cd^2+^ cations by Yb^3+^ ions, as above mentioned. The shape of the crystallization peak also evolves with increasing Yb^3+^ concentration, as can be seen in Fig. 2(a). From the SYb05 to the SYb20 sample thermograms, the peak is broadening and clearly consists of two contributions: a sharp intense one at lower temperature, and a broad weak shoulder at higher temperature. It can be assumed that the first peak is related to the crystallization of *β*-PbF_2_ crystals, as already reported in refs 23, 24, 25 while the second contribution can be associated with the formation of new crystalline phase or even phase transformation. Further investigation focused on the crystallization kinetics and identification of the crystalline phase structure would be required to fully describe the crystallization process in these SiO_2_-Al_2_O_3_-CdF_2_-PbF_2_-YF_3_-YbF_3_ glasses. Nevertheless, it is worth mentioning that the undoped sample exhibits the highest temperatures of crystallization (both onset and peak) and the largest thermal stability against crystallization (Δ*T* = 69 °C, see Fig. 2(b)). In addition, one can see in Fig. 2(a) that its crystallization peak is weaker and flatter than those on the other DSC traces, indicating therefore that the undoped glass is less prone to crystallization. Such result was expected as it is well-known that addition of RE ions like Yb^3+^ into a glassy material tends to decrease its stability vs crystallization, as previously reported in many works in the literature^23^,^24^,^26^,^27^.

As above mentioned, the knowledge of the thermo-mechanical material properties such as thermal conductivity, specific heat capacity and thermal expansion coefficient (TEC) is crucial for a proper design of an optical cooler. The heat transfer rate within the cooled material is proportional to changes in temperature, thermal conductivity and heat capacity after excitation with a suitable laser^28^. Thermo-mechanical analysis (TMA) was performed on the samples SYb02 and SYb12. The TEC determined for these samples (in the temperature range of 100-350 °C) are 11.3x10^−6^/K and 13.7x10^−6^/K, respectively. The theoretical description of the TEC has been reported for Yb^3+^-doped phosphate laser glasses elsewhere^29^. The TEC values are higher than those reported for Li_2_O–Al_2_O_3_–SiO_2_ glasses (4.6–7.5 × 10^−6^/K)^30^, phosphate glass (LiPO_3_–Al(PO_3_)_3_–Ba(PO_3_)_2_–La_2_O_3_, 9.8 × 10^−6^/K)^31^, Yb^3+^:YAG crystal (8.06 × 10^−6^/K)^32^ but lower than that of ZBLAN fluorozirconate glass (16.4 × 10^−6^/K)^33^ and comparable to that of silicate laser glasses (12.7–13.4 × 10^−6^/K)^34^. The TEC values for the investigated glasses are between those of laser cooled materials such as Yb^3+^:YAG crystal^32^ and Yb^3+^:ZBLAN glass^33^.

### Linear refractive index

**Figure 3:**
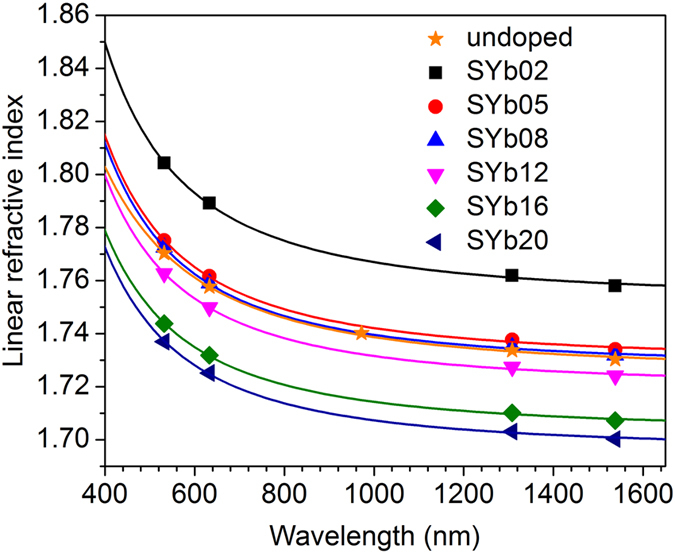
Measured linear refractive index (*n*) of the undoped and Yb^3+^-doped glasses as a function of wavelength. The data were fitted by using the Sellmeier’s dispersion formula.

The refractive indices of the glass samples were measured by the prism coupling technique with a resolution of ±0.001, and plotted in Fig. 3 as a function of wavelength and Yb^3+^ concentration. The values reported here were obtained for the transverse-electric (TE) mode of the incident laser radiation while no significant difference was observed in the transverse-magnetic (TM) mode, confirming the absence of birefringence, as expected in isotropic glass materials. First, one can observe in Fig. 3 a decrease of the refractive index with increasing the wavelength for each glass sample, showing thus their respective chromatic dispersion. The Sellmeier’s dispersion relation was used to fit the experimental data and facilitate their reading. Then, if we do not consider the undoped sample, one can observe that their refractive index decreases with increasing Yb^3+^ concentration. Such behavior is quite unusual. Indeed, glass doping with RE ions which are heavy elements compared to traditional components used to form glass (e.g. SiO_2_), generally results in increasing its refractive index. Here, the ytterbium fluoride (YbF_3_, molar mass = 230.04 g/mol) is incorporated into the glass by substituting for the cadmium fluoride, which is lighter (CdF_2_, molar mass = 150.41 g/mol), following the composition law 30SiO_2_-15Al_2_O_3_-(29-*x*)CdF_2_-22PbF_2_-4YF_3_-*x*YbF_3_ (mol%). Therefore, an increase of refractive index and density could be expected with increasing the Yb^3+^ concentration. However, while the density increase is observed with increasing Yb^3+^ concentration (as shown in Fig. S1) as expected, an opposite trend is observed for the refractive index in our glasses, as shown in Fig. 3. Interpreting the refractive index change of glasses as a function of their chemical composition is relatively complex. Indeed, it essentially depends on two factors, i.e. the glass molar volume (related to its density and molar mass) and the polarizability of its constituents. A tentative explanation can be as follows. First the high refractive index of these glasses is mainly governed by their large concentration of heavy metals with large electronic densities. Then, it is known that F^−^ anions possess a lower polarizability than O^2−^ anions^35^. The progressive replacement of CdF_2_ by YbF_3_ in these glasses implies an increase of its fluorine content to the detriment of its oxygen content, as presented in the Table 1. This results then in a decrease of the glass average polarizability. Therefore the observed decrease in glass refractive index can be ascribed here to the dominant role played by its decreasing polarizability whereas its density, which increases with increasing Yb^3+^ concentration (see Fig. S1), has a lower impact. Last, one can also notice in Fig. 3 that refractive index was accurately measured at 972 nm for the undoped sample while no value was obtained by the prism couling method at that wavelength on the Yb^3+^-doped samples. We assume that it is related to the strong absorption of Yb^3+^ ion in this spectral region. Then, the refractive index of the undoped sample (as a function of wavelength) is comprised between those of the SYb08 and SYb12 samples, illustrating once again the complexity to represent its dependence on the glass chemical composition. Following the same reasoning as above, we would have indeed expected a higher refractive index for the undoped glass than for those doped with Yb^3+^ (because of a lower fluorine content). But it is clearly not the case here as one can see in Fig. 3. It can be assumed here that the density of the undoped glass, which is significantly lower than those of the Yb^3+^-doped glasses (Fig. S1), plays a more significant role. Further structural investigation is required to elucidate such behavior.

### Electron probe micro analysis.

Electron probe micro analysis (EPMA) was carried out to identify and quantify the elemental composition of the prepared glasses. The experimental results along with the theoretical data are presented in Table 1. The synthesis process was performed at the same temperature (1100 °C) but with varying duration (1h30, 2h, 2h30, 3h, 3h30 and 4h) of the glass melting with increasing Yb^3+^ concentration. Note that the results presented in Table 1 are the mean value of five independent measurements on the same sample at different positions. It is worth mentioning that both theoretical and experimental F contents increase with increasing Yb^3+^ concentration whereas an opposite trend is observed in the case of the O content. To show the reproducibility of the synthesis process, the same glass (SYb02) was prepared three times by keeping all the conditions strictly identical (melting temperature and duration of glass melting are 1000 °C and 1 h, respectively) and the results are presented for the three samples in Table 2. The obtained maximum errors (%) in experimental results between the three samples when compared to theoretical values, indicate here an excellent repeatability of the sample preparation in the given conditions.

### Absorption spectra

**Figure 4:**
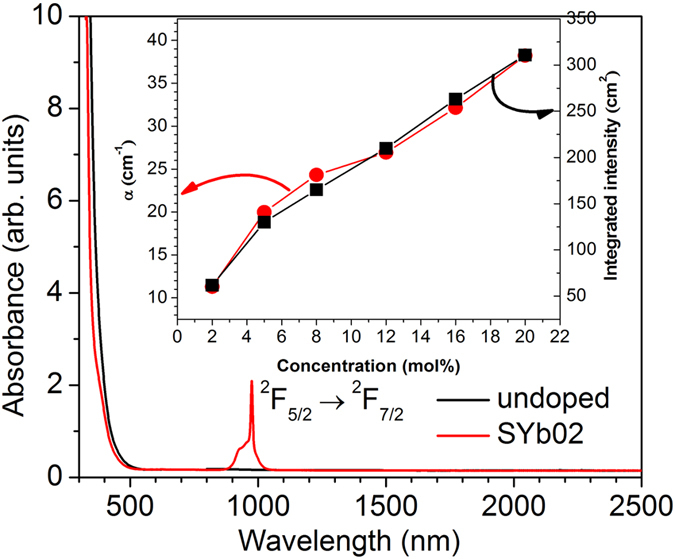
UV-visible-NIR absorption spectra of the undoped and SYb02 samples. Inset shows a variation of the linear absorption coefficient, *α* (•, at 975 nm) and the integrated absorption band intensity (■, from 900 to 1100 nm) related with the Yb^3+^ band absorption as a function of its concentration in the SYb sample.

The UV-visible-near-infrared (NIR) absorption spectra of the undoped and SYb02 samples are presented in Fig. 4, showing a broad absorption band for the SYb02 sample centered at a wavelength of 975 nm which corresponds to the Yb^3+^:^2^F_7/2_ → ^2^F_5/2_ transition. The transmission spectra obtained for the other samples (see the supplementary information, Fig. S2) show very similar profiles with the same Yb^3+^ absorption band shape, except for its intensity which depends on the Yb^3+^ concentration, as plotted in the inset of Fig. 4. The inhomogeneously broadened absorption bands are due to the electronic transitions between the Stark sublevels of the ground (^2^F_7/2_) and the excited (^2^F_5/2_) levels as well as the strong electron-phonon interaction characteristic to the glassy host^36^. The quasi-linear variation of the integrated absorption band intensity observed with increasing Yb^3+^ concentration (inset of Fig. 4) indicates the presence of a similar local environment around the Yb^3+^ ions in all the investigated glasses.

### Photoluminescence quantum yield (PLQY)

**Figure 5:**
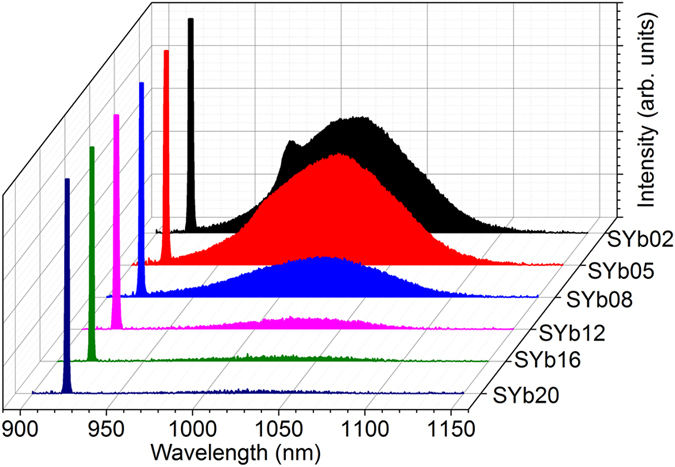
Absolute photoluminescence spectra under laser excitation at 920 nm (laser power of 510 mW) of the Yb^3+^-doped oxyfluoride glasses as a function of Yb^3+^ concentration (the sharp peak at 920 nm corresponds to the laser excitation line).

The PLQY measurements were performed inside an integrating sphere coupled to an optical spectrum analyzer (OSA) with a multimode optical fiber and then determined using the method reported in refs 22, 25. The absolute photoluminescence spectra of the samples obtained under a laser excitation at 920 nm (510 mW of power), are presented as a function of their Yb^3+^ concentration in Fig. 5. As can be seen from Fig. 5, luminescence quenching is observed for Yb^3+^ concentration higher than 2 mol%, due to either an increase in the energy transfer or reabsorption by the Yb^3+^ ions. Reabsorption or radiation trapping effects are usual when dealing with Yb^3+^-doped glasses because of the overlap of their absorption and emission bands, directly related to the Yb^3+^ ion concentration, the sample thickness (2.3 mm for our samples) and the optical path length of the photons in the medium^37^,^38^.

**Figure 6:**
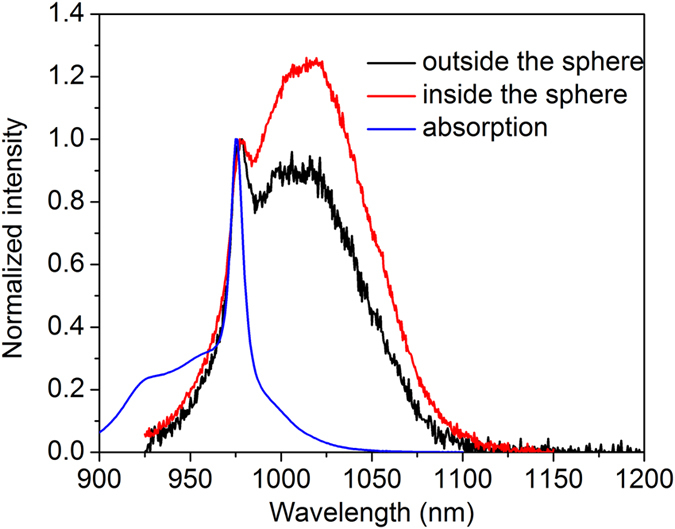
Normalized absorption and emission bands of the SYb02 sample showing their overlapping responsible for the reabsorption effect. The emission spectra were measured outside and inside the integrating sphere under laser excitation at 920 nm. Outside the sphere: the sample was excited at a depth of 1 mm from the sample surface and the luminescence was collected with a multimode fiber and measured with an OSA. Inside the sphere: the sample was excited within the integrating sphere and the absolute luminescence was collected with a multimode fiber and measured with an OSA.

**Figure 7:**
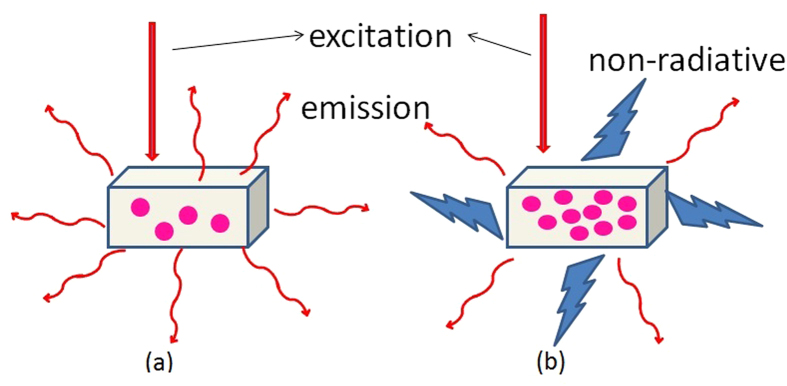
Representation of the luminescence quenching effect of Yb^3+^ ions (•) in solids at (**a**) low and (**b**) high Yb^3+^ concentration. Excitation: solid red arrow, Emission: curved red arrow, and Non-radiative transition (heat generation): zigzag.

The absorption and emission spectra of the SYb02 sample (measured inside and outside the integrating sphere) are presented in Fig. 6. As can be seen in Fig. 6, reabsorption effect is observed even for the SYb02 sample and is more predominant for samples with higher Yb^3+^ concentration (as shown in the supplementary information, Fig. S3). The emission peak position shifts towards longer wavelength and broadens due to reabsorption. The reabsorption and luminescence quenching effects observed for all the Yb^3+^ concentrations are illustrated in Fig. 7(a,b). At lower concentrations, the interaction or radiation exchange between the Yb^3+^ ions is significantly reduced and may become negligible. In Fig. 7(a), the absorbed radiation from the pump laser is re-emitted in the form of luminescence (photons) without heat generation, resulting in higher luminescence intensity. At higher concentrations, the interaction between the Yb^3+^ ions becomes stronger and their energy exchange leads to a decrease in photoluminescence intensity due to non-radiative (phonons) emission resulting in luminescence quenching effect, as schematized in Fig. 7(b). This also induces the Yb^3+^ ions luminescence reabsorption by the Yb^3+^ neighboring ions, resulting in a redshift of the emission band.

PLQY is evaluated by using both Eqs (2) and (5), giving similar values for each SYb sample as a function of Yb^3+^ concentration, as summarized in Table 1. The standard deviation of measurements is around ±0.11, which is typical of absolute PLQY measurements. Moreover, the acquisitions were repeated 5 times to ensure the consistency of the results. The highest PLQY value (0.99) was obtained for the SYb02 sample. Further increase of the Yb^3+^ concentration results in a PLQY decrease, owing to the concentration quenching effect. Then, the PLQY obtained for the SYb02 sample is comparable to that of Yb^3+^:YAG single crystal (containing 3 at.% of Yb^3+^)^12^ and Yb^3+^:ZBLANP glass^6^ in which optical cooling has been already demonstrated. It is worth mentioning that a high PLQY close to unity is one of the most important conditions in order to achieve a better cooling efficiency by removing successfully heat from the sample in every cooling cycle^6^. The background absorption coefficient (*α*_b_) of the samples, measured with a 1300 nm wavelength laser by the calorimetric method described in our earlier works^12^,^25^ is reported in Table 3. One observes a background absorption increase with increasing Yb^3+^ concentration.

The mean fluorescence wavelength (*λ*_f_) is calculated by using Eq. (4). The laser cooling/reduced heating can be expected when the samples are excited at or above *λ*_f_^6^. As can be seen from Table 3, the *λ*_f_ value increases with increasing Yb^3+^ concentration due to reabsorption. The *λ*_f_ is found to be 1003(1) nm for the SYb02 sample which is larger than that reported for the Yb^3+^:ZBLANP (995 nm, 1 wt% of Yb^3+^)^6^. Hence, as the SYb02 sample exhibits high PLQY and low background absorption when compared with the other investigated samples, it appears to be the best candidate for laser cooling application besides serving as a reference sample for PLQY measurements in the near-infrared region.

### Decay curves

**Figure 8:**
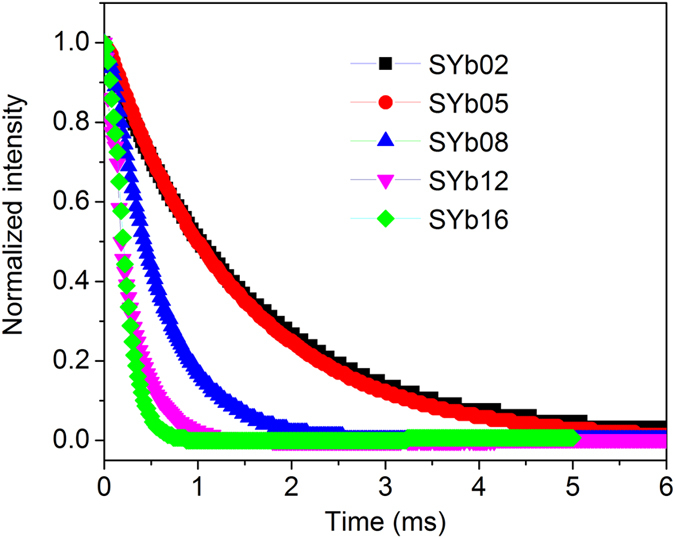
Decay curves for the Yb^3+^: ^2^F_5/2_ → ^2^F_7/2_ transition of the SYb samples as a function of Yb^3+^ concentration, under laser excitation at 940 nm.

The luminescence decay curves of the Yb^3+^:^2^F_5/2_ → ^2^F_7/2_ transition were measured by exciting with 940 nm wavelength laser and monitoring above the 975 nm wavelength emission, as shown in Fig. 8. The luminescence lifetime (*τ*) of the Yb^3+^:^2^F_5/2_ excited level was evaluated from a single exponential fit. It is observed that the *τ* of the Yb^3+^:^2^F_5/2_ excited state shortens from 1.52 to 0.19 ms in the investigated glasses when Yb^3+^ concentration increases from 2 mol% to 20 mol%. These results indicate that the decrease in PLQY with increasing Yb^3+^ concentration is not only due to reabsorption but also to concentration quenching. The quenching of lifetime may be either due to multiphonon relaxation, energy transfer among the Yb^3+^ ions (diffusion limited)^39^ or direct coupling with OH^−^ groups^37^. In the present study, since the amount of OH^−^ groups is expected to be relatively constant in all the samples, it is assumed that the most dominant mechanisms for lifetime quenching are the energy transfer among the Yb^3+^ ions, as well as the multiphonon relaxation. The longest lifetime measured here is 1.52 ms for the SYb02 sample, which is longer than that reported for the Yb^3+^:YAG crystal (1.1 ms, for a concentration of 2.5 at.% Yb^3+^)^40^ but shorter than that measured for the Yb^3+^:ZBLAN glass (1.82 ms, for a concentration of 2 mol% Yb^3+^)^41^. The high PLQY, which is a key parameter for laser cooling process, of the SYb02 sample with lower Yb^3+^ concentration (2 mol%) indicates its higher potential for laser cooling. Longer lifetime is not an obstacle for cooling, but it is not desirable, since it can slow down the cooling process.

### Pump power dependence PLQY and lifetime studies

**Figure 9:**
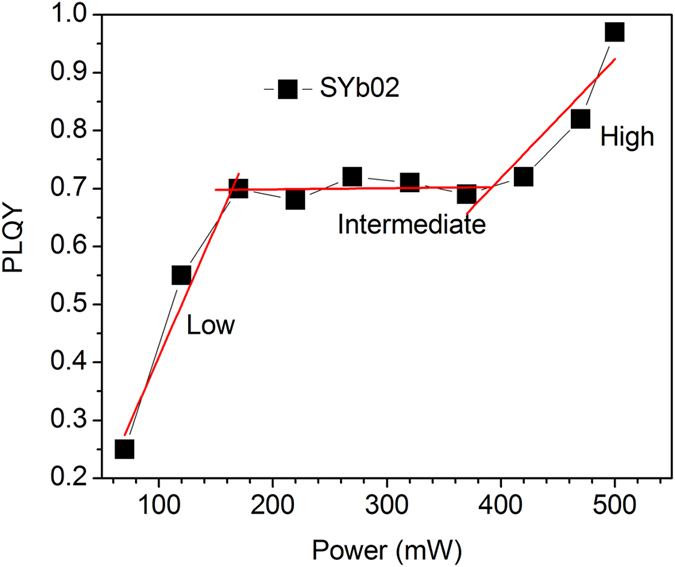
Variation of PLQY with pump power for the 2 mol% Yb^3+^-doped glass.

The pump power dependence of PLQY and lifetime measurements were performed on the Yb^3+^-doped glasses. Boconilli *et. al.* have reported on the pump power dependence studies of upconversion (UC, process consisting in the absorption of two or more photons of low energy followed by the emission of one photon of higher energy) PLQY in Er^3+^:*β*-NaYF_4_ nanocrystals by considering the effect of reabsorption for solar cell applications^42^. This is the first time to the best of our knowledge that the pump power dependence PLQY of Yb^3+^-doped glasses for laser cooling prospective is reported by considering. The PLQY (and the intensity of NIR emission as well) always follows a linear dependence with the pump power in our Yb^3+^-doped oxyfluoride glasses, as presented in Fig. 9. The PLQY was found to be as high as 0.99 for 510 mW of laser power measured at the entrance of the integrating sphere. Three regions are distinguished in Fig. 9, the first region for the low powers, the second one for intermediate powers and the third one for high excitation powers. The PLQY follows a progressive increase at low excitation powers whereas it remains unchanged at the intermediate excitation powers. This can be explained by the fact that there is no influence of the absorbed power by the Yb^3+^ ions which means that reabsorption may play a crucial role for this behavior providing a low fluorescence escape efficiency. At higher powers, the PLQY progressively increases due to the enhanced absorption of Yb^3+^ ions within the unit area for a fixed concentration. Moreover, the bleaching occurring at high pump powers can decrease significantly reabsorption, inducing further increase in PLQY^43^. The upconversion effects from the Tm^3+^ and Er^3+^ ions present as impurity traces (detailed discussion in the next section) at high pump powers may also cause a slight deviation from a straight line.

**Figure 10:**
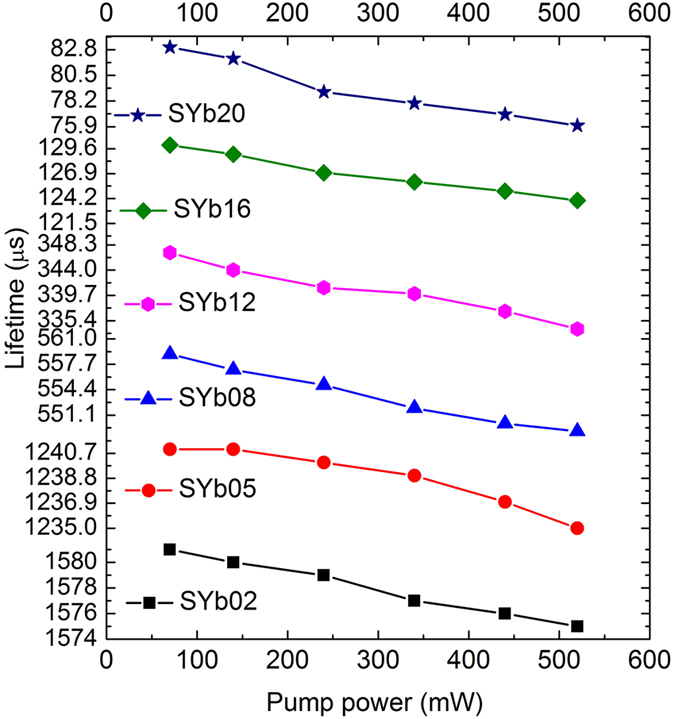
Power dependence of lifetime for SYb samples with different Yb^3+^ concentrations.

The power dependence of lifetime for the Yb^3+^-doped glasses is shown in Fig. 10. As can be seen in Fig. 10, there is a small but consistent decrease of lifetime with increasing pump power, especially at higher Yb^3+^ concentration (20 mol%). This may be due to either a decrease in the lifetime and PLQY because of lower reabsorption due to bleaching effects^43^. The decrease in lifetime (Fig. 10) indicates that non-radiative and also upconversion processes should play an important role which means the non-radiative rate, *W*_nr_, increases with increasing the Yb^3+^ concentration. The most important channels on the non-radiative rate increase are energy migration among Yb^3+^ ions, followed by transfer to impurity centers: trapping by defects such as OH^−^ radicals, radiation trapping of energy among Yb^3+^ ions, interaction between Yb^3+^ ions and the glassy host defects.

### Upconversion luminescence

**Figure 11:**
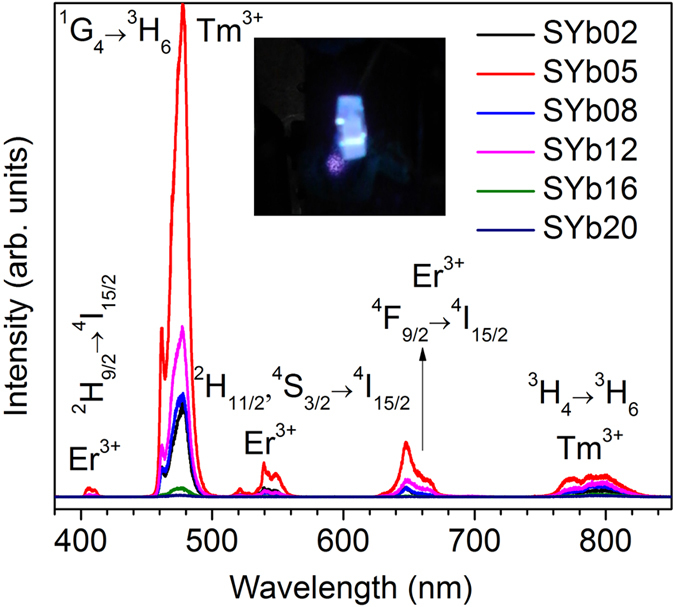
UC emission spectra of Er^3+^ and Tm^3+^ ions in the SYb samples under laser excitation at 975 nm with 80 mW laser power as a function of Yb^3+^ concentration. Inset is a picture of the bright blue UC emission of the SYb05 sample coming from the presence of Tm^3+^ ions as an impurity.

The UC emission spectra of glasses recorded under laser excitation at 975 nm (80 mW power) are shown in Fig. 11 while a photograph of the UC luminescence from the SYb05 sample is shown in inset. It is clear that a higher UC intensity at 478 nm is obtained for the SYb05 sample than for the other samples. All the samples exhibit UC emissions at 478 nm (^1^G_4_ → ^3^H_6_) and 800 nm (^3^H_4_ → ^3^H_6_) originating from Tm^3+^ impurity as well as 410 nm (^2^H_9/2_ → ^4^I_15/2_), 539 nm (^2^H_11/2_, ^2^S_3/2_ → ^4^I_15/2_) and 647 nm (^4^F_9/2_ → ^4^I_15/2_) originating from Er^3+^ ions also present as an impurity. Those ions are excited thanks to energy transfer (Addition of Photons by Transfer of Energy: APTE effect) from the Yb^3+^ ions which act as a sensitizer^44^. It was evidenced that the APTE effect is 10^5^ times more efficient than the cooperative luminescence which is usual in Yb^3+^-doped samples at high concentrations for the same Yb^3+^-Yb^3+^ distances^45^,^46^. Due to this reason no cooperative luminescence was observed in these glasses even at high Yb^3+^ concentration. The pump power dependence of the UC luminescence is shown in Fig. S4. It is worth mentioning that no UC emission was observed from the Yb^3+^ free sample (as shown in Fig. S5). Therefore, it is clear that these Tm^3+^ and Er^3+^ traces (contents of respectively less than 10 and 8 ppm according to the certificate of analysis provided by the chemical supplier) originate from the YbF_3_ starting powder, in spite of its relatively high purity (99.99%). Complete separation of RE ions during their manufacturing process to obtain ultra-high purity raw materials is indeed a well-known issue in the industry.

## Conclusion

Heavily Yb^3+^-doped 30SiO_2_-15Al_2_O_3_-(29-*x*)CdF_2_-22PbF_2_-4YF_3_-*x*YbF_3_ (where *x* = 2, 5, 8, 12, 16 and 20 mol%) oxyfluoride glasses have been fabricated and characterized from a thermal and spectroscopic point of view. Their glass transition and crystallization temperatures as well as thermal expansion coefficient were determined by thermal analysis. The measured linear refractive index of the SYb samples varies from 1.780 to 1.730 at 532 nm and decreases with increasing Yb^3+^ concentration. Luminescence intensity at 1020 nm under laser excitation at 920 nm decreases with increasing the Yb^3+^ concentration. The highest photoluminescence quantum yield (0.99, near unity) was obtained for the 2 mol% Yb^3+^-doped sample and was then found to decrease when increasing the Yb^3+^ concentration. The PLQY increases with increase in the pump power up to 510 mW, the limit of the laser used in this work. The mean fluorescence wavelength was evaluated from the emission spectrum and reported to be 1003(1) nm for the 2 mol% Yb^3+^: glass which increases with increasing Yb^3+^ ion concentration. Pump power dependence studies have revealed a linear increase in the PLQY and a decrease in the lifetime with increasing the pump power. The lifetime of the ^2^F_5/2_ level shortens from 1.52 ms to 0.19 ms with increasing the Yb^3+^ concentration.

The 2 mol% Yb^3+^-doped oxyfluoride glass with its high PLQY, its low maximum phonon energy and low background absorption is the most promising candidate for laser cooling and solid-state laser applications besides serving as a reference to calibrate the instruments for PLQY measurements. Future works will focus on one hand on the fabrication of glasses of ultra-high purity, which is a required condition to achieve optical cooling and; on the other hand, on the preparation of ultra-transparent nano-glass-ceramics in a similar vitreous system with the aim to further enhance the photoluminescence quantum yield, to decrease the background absorption and *in fine* to demonstrate laser cooling in this material.

## Methods

Oxyfluoride glasses with chemical composition 30SiO_2_-15Al_2_O_3_-(29-*x*)CdF_2_-22PbF_2_-4YF_3_-*x*YbF_3_ (mol%), where *x* = 2, 5, 8, 12, 16 and 20 were prepared by the conventional melt-quenching technique. The glass samples were labeled as SYb02, SYb05, SYb08, SYb12, SYb16 and SYb20, respectively. High purity commercial starting materials (Aldrich, 99.99%) were thoroughly mixed in an agate mortar and then loaded into a platinum crucible. The powders were then melted and homogenized at 1100 °C for different durations (1h30, 2h, 2h30, 3h, 3h30 and 4h with increasing Yb^3+^ concentration respectively) in a muffle furnace under ambient atmosphere in the covered crucible. The glass melt was then casted into a stainless steel mold preheated close to the glass transition temperature (*T*_*g*_), subsequently annealed at the same temperature for 5 h and slowly cooled to room temperature to remove any residual internal stress. The glass samples were finally polished to optical quality for further characterization.

The density of the samples was measured by using a Mettler Toledo XSE204 densimeter with a resolution of ±0.001 g/cm^3^. The linear refractive index of the samples was measured by employing the prism coupling technique (Metricon 2010) at different wavelengths, 532, 633, 972, 1308 and 1538 nm. Differential scanning calorimetric (DSC) measurements were performed by using a Netzsch DSC Pegasus 404F3 apparatus on glass pieces in PtRh pans at a heating rate of 10 °C/min. The thermal expansion coefficient (TEC) was measured by using a Netzsch TMA 402F1 Hyperion thermo-mechanical analyzer apparatus on glass rods of 5 mm length and 10 mm diameter at a heating rate of 5 °C/min up to the glass softening point with a load of 0.02 N. The TEC of the sample was then determined in the temperature range from 100 to 300 °C. UV-visible-near infrared transmission spectra were recorded on a Cary 5000 (Varian) double-beam spectrophotometer. The photoluminescence quantum yield (PLQY) of the samples (10 mm × 2 mm × 2 mm) was measured by pumping at a wavelength of 920 nm with a Ti:sapphire laser, collecting the emitted light from an integrating sphere (2″) (Thorlabs IS200) and coupling it through a multimode optical fiber to an optical spectrum analyzer (OSA) operating in the wavelength range of 800–1600 nm. The photoluminescence spectra were also measured outside the integrating sphere under 920 nm wavelength laser excitation. The upconversion luminescence spectra were recorded using a Nanolog (Horiba Jobin Yvon) fluorimeter equipped with a double monochromator and a photomultiplier tube sensitive from 250 to 825 nm. A laser diode operating at 975 nm coupled with a standard single-mode pigtailed fiber was used to excite the samples after beam collimation and focusing on the sample surface with a lens (*f* = 50 mm). Decay curves were recorded with a resolution of 10 μs by using a photodiode (Thorlabs SM05PD1B). The signal was amplified by a bench top transimpedance amplifier (Thorlabs PDA200C) and read with a digital storage oscilloscope (Tektronix TDS2012CB 100MHZ 2GS/s).

The pump power dependence PLQY studies were performed by exciting the samples with a 920 nm wavelength laser from a Ti: Sapphire while the output power was maintained constant by using a Glan-Thompson polarizer. Part of the laser power was tapped by a glass slide and monitored with a Keithley 6487 Picoammeter. The transmitted beam was focused at the entrance port of the integrating sphere and directed to the center of the sphere where the sample was situated. The diffused light from the sphere walls was collected by a multimode fiber of 200 μm diameter and detected with an Ando AQ6317B optical spectrum analyzer. The data was collected and processed in a computer which measures 50 spectra, while normalizing them to the tapped optical power. All the measurements were performed at room temperature.

## Additional Information

**How to cite this article**: Krishnaiah, K. V. *et al.* Development of ytterbium-doped oxyfluoride glasses for laser cooling applications. *Sci. Rep.* **6**, 21905; doi: 10.1038/srep21905 (2016).

## Supplementary Material

Supplementary Information

## Figures and Tables

**Table 1:** Elemental quantitative analysis (EPMA) of the undoped and Yb^3+^-doped samples compared with the theoretical values.

Sample	Elements (at.%)															
	Si		Al		Pb		Cd		Y		Yb		O		F	
	Theo.	Exp.	Theo.	Exp.	Theo.	Exp.	Theo.	Exp.	Theo.	Exp.	Theo.	Exp.	Theo.	Exp.	Theo.	Exp.
Undoped	9.091	n/a	9.091	n/a	6.667	n/a	8.788	n/a	1.212	n/a	0	n/a	31.818	n/a	33.333	n/a
SYb02	8.929	7.516	8.929	8.161	6.548	8.028	8.036	10.633	1.190	1.507	0.595	0.872	31.250	40.027	34.524	23.256
SYb05	8.850	7.440	8.850	7.938	6.490	8.056	7.080	9.901	1.180	1.488	1.475	1.864	30.973	39.663	35.103	23.650
SYb08	8.772	7.854	8.772	6.443	6.433	8.271	6.140	9.458	1.170	1.562	2.339	3.112	30.702	39.124	35.673	24.175
SYb12	8.671	7.766	8.671	7.154	6.358	7.696	4.913	5.976	1.156	1.487	3.468	5.886	30.347	36.333	36.416	27.702
SYb16	8.571	7.822	8.571	7.126	6.286	7.655	3.714	5.953	1.143	1.479	4.571	5.924	30.000	36.632	37.143	27.409
SYb20	8.475	7.568	8.475	7.557	6.215	7.400	2.542	4.270	1.130	1.405	5.650	7.215	29.661	35.830	37.853	28.755

**Table 2:** Elemental quantitative analysis (EPMA) of three SYb02 samples prepared under identical conditions.

Elements	Theoretical, at.%	Sample 1Exp., at.%	Sample 2Exp., at.%	Sample 3Exp., at.%	Max. error betweenprepared samples, %
Si	8.929	7.508	7.599	7.585	1.1
Al	8.929	8.921	8.751	8.873	1.7
Pb	6.548	7.803	7.771	7.681	1.2
Cd	8.036	9.596	9.685	9.646	0.9
Y	1.190	1.314	1.274	1.295	4.0
Yb	0.595	0.690	0.717	0.728	3.8
O	31.250	38.693	38.464	38.679	2.3
F	34.524	25.475	25.740	25.513	2.6

**Table 3:** Photoluminescence quantum yield (PLQY) of the Yb^3+^-doped samples is determined by using the Eqs (2) and (5), pump wavelength (*λ*_P_), limiting wavelength (*λ*_cutoff_) which separates the integration of *N*_ipj_ and *N*_epj_ (*j* = *a*,*b* and *c*), mean fluorescence wavelength (*λ*_f_) which takes into account reabsorption, background absorption (*α*_b_) determined at 1300 nm laser by using the calorimetry method described in refs 12,25.

Sample	*λ*_P_ (nm)	PLQY (±0.11)		*λ*_cutoff_ (nm)	*λ*_f_ (nm)	*α*_b_ (m^−1^)
		Eq. (2)	Eq. (5)			
3 at.% Yb^3+^:YAG^12^	920	0.99	1.02	924	1010(1)	0.28
SYb02^25^	920	0.99	1.00	924	1003(1)	1.27
SYb05	920	0.51	0.42	924	1005(1)	1.64
SYb08	920	0.13	0.12	924	1006(1)	2.29
SYb12	920	0.11	0.13	924	1008(2)	–
SYb16	920	0.07	0.05	924	1012(2)	–
SYb20	920	0.01	0.01	924	1019(3)	–
