# Supplementary material for: Development of ytterbium-doped oxyfluoride glasses for laser cooling applications
Source: Sci Rep. 2016 Feb 26;6:21905. doi: 10.1038/srep21905 (PMC4768141; doi:10.1038/srep21905)
Supplement: Supplementary Information [file srep21905-s1.doc]

**Supplementary information:**

**Development of ytterbium-doped oxyfluoride glasses for laser cooling applications**

**Kummara Venkata Krishnaiah^1,2,*^, Elton Soares de Lima Filho^1^, Yannick Ledemi^2^, Galina Nemova^1^, Younes Messaddeq^2^ and Raman Kashyap^1,3^**

*^1^Department of Engineering Physics, Polytechnique Montréal, 6079, Station Centre-ville, Montréal H3C 3A7, Canada*

*^2^Centre d'Optique, Photonique et Laser, 2375 Rue de la Terrasse, Université Laval, Québec G1V 0A6, Canada*

*^3^Department of Electrical Engineering, Polytechnique Montréal, 6079, Station Centre-ville, Montréal H3C 3A7, Canada*

*^*^Corresponding author: venkata-krishnaiah.kummara@polymtl.ca*

***Density***

The density of the sample increases with increasing Yb^3+^ concentration as shown in Fig. S1. The results shown in Fig. S1 are the mean values of four independent measurements. The high density of the samples is due to the presence of heavy metal ions in the glass matrix. A lower value is recorded for the undoped sample when compared to the Yb^3+^-doped samples.

***
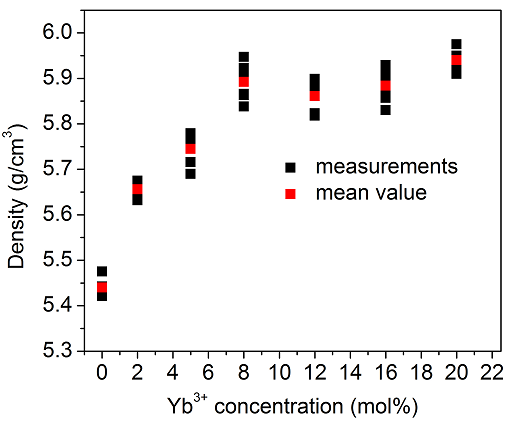
***

**Fig. S1.** Variation of density for the undoped and Yb^3+^-doped samples as a function of Yb^3+^ concentration in the 30SiO_2_‒15Al_2_O_3_‒(29-*x*)CdF_2_‒22PbF_2_‒4YF_3_‒*x*YbF_3_ glasses.

***Transmission spectra***

The transmission spectra of undoped and different Yb^3+^-doped samples (SYb02, SYb05 and SYb08) are shown in Fig. S2. The absorption band related to Yb^3+^ is increases with increasing its concentration (only from 2 mol% to 8 mol%), as expected. The transmission spectra recorded on the samples of same thickness with larger concentration of Yb^3+^ ions are not presented here as they are showing a saturation effect of the Yb^3+^ absorption around 980 nm. Note that the step observed at 800 nm corresponds to the change of detector gratings of the spectrophotometer during acquisition.

**
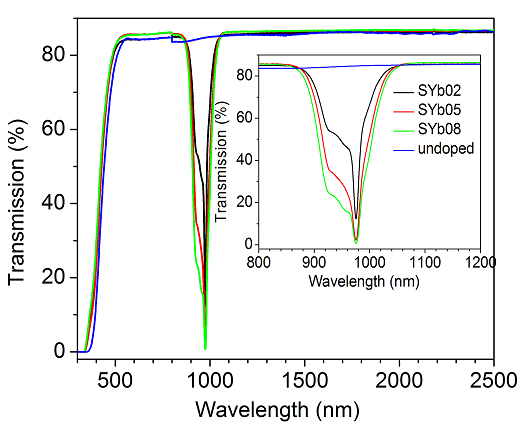
**

**Fig. S2**. Transmission spectra of the undoped and Yb^3+^-doped samples with 2.3 mm thickness. Inset shows the magnified region of the Yb^3+^ absorption band.

***Reabsorption effects***

The PL spectra of the samples shown in Fig. S3 were measured at the surface and a depth of 1 mm from the surface. First, the luminescence intensity decreases with increasing Yb^3+^ concentration. Then, the reabsorption effect is clearly observed and it is predominant at higher Yb^3+^ concentration when compared to lower concentrations. The emitted photons must escape from the sample to achieve laser cooling. If the reabsorption effect is high, or if the fluorescence photons undergo a total internal reflection, one can expect the heating of the samples. In samples with very low Yb^3+^ concertation the cooling process is very weak. Future work will focus on identifying samples with optimized Yb^3+^ concentration exhibiting negligible reabsorption effect in order to leads a high cooling power.


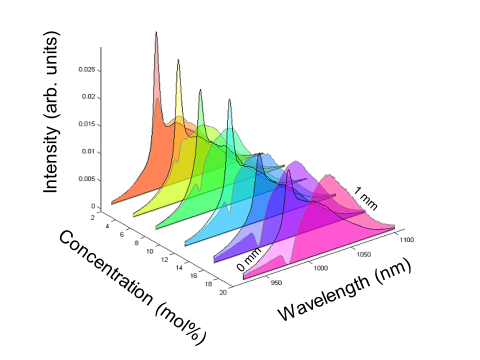


Fig. S3. Photoluminescence of the SYb samples recorded at the surface (0 mm) and at a depth of 1 mm from the surface as a function of Yb^3+^ concentration upon laser excitation at 920 nm. The spectra overlapping for each sample shows the reabsorption effects.

***Upconversion emission***

The upconversion (UC) emission intensity dependence on the pump power at 975 nm was studied to identify the UC involved mechanisms for the SYb05 sample (this sample was selected as it exhibits the highest UC emission intensity). The spectra recorded for different pump powers are shown in Fig. S4. One can observe the UC emissions from Tm^3+^ and Er^3+^ impurities that were excited through the energy transfer process after an efficient excitation of Yb^3+^ ions at 975 nm. The UC emission intensity from Tm^3+^ increases with increasing the pump power and the log-log plot of this dependence is shown in the inset of Fig. S4. A similar behavior is also observed for the Er^3+^ ion. From the slope, it is clear that two NIR pump photons are required for UC blue emission process.

**
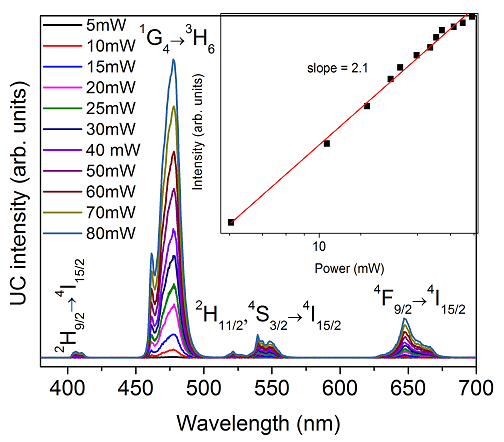
**

**Fig. S4**. Power dependence UC emission spectra of Er^3+^ and Tm^3+^ ions in SYb05 sample upon 975 nm laser excitation. Inset shows the log-log plot of blue (Tm^3+^) emission intensity as a function of laser excitation power.

To identify the origin of Tm^3+^ and Er^3+^ impurities in our samples, suspected to coming from the YbF_3_ commercial starting powder, the PL measurements were also performed on the Yb^3+^ free sample by laser diode excitation at 975 nm with a power of 25 mW. The obtained UC emission spectra of undoped and SYb05 samples are compared in Fig. S5. A magnification of the spectral region of interested is also presented in inset of Fig. S5. One can conclude here that our samples contamination with Tm^3+^ and Er^3+^ traces comes from the utilized YbF_3_ starting materials employed. The concentration of these impurities is expected to increase with increasing the sample Yb^3+^ concentration.

**
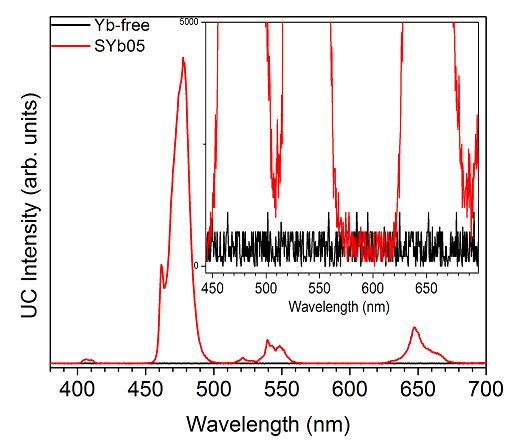
**

**Fig. S5**. UC emission spectra of Er^3+^ and Tm^3+^ ions in the undoped (black) and SYb05 (red) samples, under laser excitation at 980 nm with a power of 25 mW. Inset shows the magnification of the region of interest for a better comparison.
